# Understanding gut microbial diversity using systems based on the Constrained-Disorder Principle provides a novel approach to targeting gut microbiome therapies

**DOI:** 10.3389/fmicb.2025.1713775

**Published:** 2025-12-10

**Authors:** Ofer Perzon, Yaron Ilan

**Affiliations:** Hadassah Medical Center, Faculty of Medicine, Hebrew University, Jerusalem, Israel

**Keywords:** digital health, variability, gut diversity, microbiome, artificial intelligence

## Abstract

**Background/aims:**

The diverse composition of the gut microbiome is vital for human health, influencing digestion, immune regulation, and disease resistance. While higher diversity is generally associated with resilience, reduced and excessive diversity can lead to health issues.

**Methods:**

This paper introduces the Constrained Disorder Principle (CDP) as a new framework for understanding microbial diversity.

**Results:**

The CDP emphasizes the significance of maintaining variability within certain boundaries to sustain ecosystem stability and promote health. It considers intra- and inter-individual variability, illustrating how microbial ecosystems adapt throughout different life stages, genetic backgrounds, and environmental exposures. Integrating CDP-based artificial intelligence systems may enable the establishment of personalized diversity thresholds, predict dysbiosis, and refine interventions such as probiotics, prebiotics, fecal microbiota transplantation, and customized dietary strategies. CDP-driven platforms enhance therapeutic precision by utilizing variability induction, feedback loops, and microbial signature analysis to optimize diversity goals and identify actionable biomarkers.

**Conclusion:**

This platform can pave the way for adaptive, individualized disease prevention and treatment strategies, bridging the gap between microbial ecology and precision medicine. It provides a powerful tool for harnessing the therapeutic potential of gut microbial diversity to enhance human health.

## Introduction

The diversity of the gut microbiome is generally seen as beneficial for overall health. A diverse microbiome is associated with a stronger and more resilient digestive system, enhanced immune function, and improved health outcomes. However, there is no one-size-fits-all answer regarding how much diversity is considered “too much” or whether there is an upper limit that could lead to disease. The relationship between microbial diversity and health is complex and influenced by various factors, including diet, genetics, age, medications, and environmental exposures. Although a diverse microbiome is generally linked to good health, specific changes in diversity—whether increases or decreases—have been associated with a range of health conditions (Hou et al., 2022). The challenge lies in defining “optimal” or “healthy” microbial diversity and understanding how these boundaries may change across an individual’s lifespan or during disease states.

The Constrained Disorder Principle (CDP) offers a novel perspective on understanding microbial diversity. It defines biological systems by their inherent variability, suggesting that a certain level of disorder is essential for these systems to function effectively and adapt to changes. In this context, variability is not merely tolerated; ecosystems must maintain stability and resilience in the face of environmental pressures (Ilan, 2022a).

This paper reviews key concepts related to gut diversity, emphasizing its dynamic boundaries and exploring how the CDP can serve as a framework for optimizing gut microbial diversity. It highlights the potential of CDP-based artificial intelligence (AI) systems to redefine therapeutic strategies, personalize interventions, and offer more precise control over microbial ecosystems. Ultimately, this approach could pave the way for more effective microbiome-targeted therapies, improving health outcomes across various conditions.

## The complexity of gut microbial diversity

The human gut microbiome is a complex and dynamic ecosystem that plays a crucial role in both health and disease. It consists of trillions of microorganisms, including bacteria, viruses, fungi, and archaea, that interact in intricate networks to shape the physiology of their hosts. This ecosystem exhibits considerable variability between individuals and populations, reflecting a complex interplay of genetic, environmental, and lifestyle factors (Huttenhower et al., 2012). Temporal intra-individual variability is a significant characteristic, as there are considerable fluctuations in the abundance of most major gut genera on a daily basis. Studies show that 78% of microbial genera exhibit more considerable variability in their absolute abundance within individuals than between individuals, with shifts of up to 100-fold occurring over a six-week period (Vandeputte et al., 2021).

Numerous extrinsic and intrinsic factors influence individual variation in the gut microbiome, including diet, lifestyle, medication use, genetics, and geographic location (Tap et al., 2023). The physiology of the gut and environmental factors, including transit time, nutrient availability, stool moisture, and pH levels, significantly modulate intra- and inter-individual variations in the composition and metabolism of the gut microbiome (Procházková et al., 2024).

Understanding the complexity of the gut microbiome is essential for recognizing its adaptive nature. Variability is not merely a characteristic but a necessary condition for the microbiome’s resilience. This resilience enables the microbiome to respond effectively to changes in diet, infections, and various stressors. This dynamic adaptability aligns with the CDP, which posits that a system’s ability to fluctuate within defined limits is crucial for maintaining stability (Ilan, 2022a).

## The Constrained-Disorder Principle explains physiological diversity and offers a framework to utilize it for overcoming malfunctions

The Constrained Disorder Principle (CDP) suggests that biological systems function within a framework of limited variability, where disorder is kept within dynamic boundaries (Ilan, 2023b,c; Ilan, 2024d). This principle is essential for understanding the random nature of biological processes, going beyond the idea of biological relativity and physiological regulatory networks. The CDP highlights the vital role of randomness at various levels of biological organization, ranging from the molecular to the systemic level. This inherent variability enables biological systems to adapt dynamically to environmental pressures, physiological demands, and internal disturbances, thereby ensuring survival and success in changing conditions (Andermann et al., 2022; Herrera et al., 2023; Hurvitz and Ilan, 2023; Ilan, 2020a; Ilan, 2021a; Ilan, 2022b; Ilan, 2023c; Ilan, 2025; Jurasinski et al., 2009; Magne et al., 2020; Pinart et al., 2022; Rinninella et al., 2019; Sigawi and Ilan, 2023; Sigawi et al., 2023; Thukral, 2017).

This randomness is not mere noise; instead, it is a fundamental feature that underpins the adaptability and efficiency of biological systems. Random fluctuations in gene expression, protein interactions, and microbial populations enable living systems to explore a variety of functional states. This flexibility can provide evolutionary advantages or help organisms recover from disruptions. According to the CDP, this inherent variability is a built-in corrective mechanism. It allows living systems to dampen, compensate for, or even utilize deviations from homeostasis to enhance overall functionality. The principle suggests that living systems thrive on an optimal level of variability—too little leads to rigidity and vulnerability, while too much results in instability and dysfunction (Ilan, 2022a). This concept is particularly relevant in AI, where algorithms based on the CDP can improve system functionality by integrating variability (Hurvitz and Ilan, 2023; Ilan, 2020a; Ilan, 2021a; Ilan, 2022a,b; Ilan, 2024e; Ilan, 2025; Sigawi and Ilan, 2023; Sigawi et al., 2023).

In the context of the gut microbiome, the CDP provides a framework to assess diversity and stability, emphasizing how microbial ecosystems achieve resilience and adaptability while promoting host health.

## Microbiome and variability: alpha and beta diversity in healthy and diseased states

Alpha diversity refers to the variety of species within a specific ecosystem, usually measured by the number of species present (species richness). Beta diversity, on the other hand, assesses the differences in species diversity between multiple ecosystems, highlighting species turnover and offering insights into the variability and connectivity of these ecosystems. In simpler terms, alpha diversity refers to the richness and evenness of microbial communities within a single individual, whereas beta diversity examines the differences in microbial compositions between individuals. It sheds light on how external influences and unique traits of the host shape the microbiome (Andermann et al., 2022; Herrera et al., 2023; Jurasinski et al., 2009; Thukral, 2017).

The predominant phyla in the human gut microbiome are Firmicutes and Bacteroidetes, which collectively account for the majority of the bacterial population. Other notable phyla include Proteobacteria, Actinobacteria, Verrucomicrobia, and Fusobacteria. At the genus level, some of the most commonly identified genera are Bacteroides, Faecalibacterium, Escherichia/Shigella, Sutterella, Akkermansia, Parabacteroides, Prevotella, Lachnoclostridium, Alistipes, Fusobacterium, and several members of the Lachnospiraceae family. These genera play crucial roles in various functions, including digestion, immune modulation, and maintaining gut homeostasis (Belizário and Napolitano, 2015; Jiao et al., 2022; Magne et al., 2020; Pinart et al., 2022; Rinninella et al., 2019).

Changes in dominant bacterial groups can indicate dysbiosis, an imbalance in the microbial community, and may suggest a higher risk of disease. Healthy individuals typically exhibit moderate to high alpha diversity, characterized by a diverse range of microbial species. In contrast, reduced diversity is often seen in multiple disease states, including inflammatory bowel disease (IBD), Parkinson’s disease, chronic obstructive pulmonary disease (COPD), liver disease, *Clostridium difficile* infections, urinary stone disease, obesity, diabetes, cardiovascular disease, cerebral small vessel disease, and Meniere’s disease, among others (Duvallet et al., 2017; Ma et al., 2019). Although the loss of microbial diversity is frequently observed in various diseases, the mechanisms and causal relationships underlying this phenomenon remain complex and multifactorial.

While low alpha diversity is often associated with disease, it is not a universal indicator of poor health. Surprisingly, some conditions, such as cervical cancer and some instances of autism spectrum disorder, show increased diversity (Chen et al., 2024; Sims et al., 2019). This suggests that the role of the microbiome in disease is highly context-dependent and may involve changes in specific taxa, rather than overall diversity alone.

Table 1 presents examples of disease conditions associated with changes in microbial diversity.

**Table 1:** Disease conditions linked to alterations in microbial diversity.

Diversity change	Associated disease states
Decreased diversity	Inflammatory Bowel Disease (IBD) Parkinson’s Disease. Chronic Obstructive Pulmonary Disease (COPD). Chronic Liver Disease. *Clostridium difficile* Infections. Urinary Stone Disease. Obesity. Diabetes. Cardiovascular Disease. Cerebral Small Vessel Disease. Meniere’s Disease (Shi et al., 2022; Manor et al., 2020).
Increased diversity	Cervical Cancer. Autism Spectrum Disorder (Sims et al., 2019; Chen et al., 2024).

The microbiome has a significant influence on children’s development. Unlike adults, who typically have high levels of alpha diversity, children are not born with this same level of diversity. Instead, they gradually acquire it over time. This development process appears to be essential and offers some protective benefits. However, having high diversity alone is not sufficient; a community that does not provide support for its diverse members cannot be sustained (Niu et al., 2020; Ovaska et al., 2024; Stewart et al., 2018).

Associations were identified between the gut microbiota and approximately 150 host phenotypic features across around 3,400 individuals. The primary axes of taxonomic variance in the gut were described, revealing a potential diversity peak along the Firmicutes-to-Bacteroidetes axis. Additionally, associations were discovered between microbiome composition, host clinical markers, and lifestyle factors, both known and unknown. These included host-microbe associations specific to certain compositions (Manor et al., 2020; Shi et al., 2022).

The Human Microbiome Project has analyzed the largest cohort and the most distinct, clinically relevant body habitats. The diversity and abundance of signature microbes in each habitat vary significantly, even among healthy individuals, indicating substantial niche specialization both within and among individuals. The project identified an estimated 81–99% of the genera, enzyme families, and community configurations present in the healthy Western microbiome. Despite variations in community structure, the metagenomic presence of metabolic pathways remained stable among individuals (Huttenhower et al., 2012). Additionally, ethnic and racial background emerged as one of the strongest associations with microbial pathways and clinical metadata. The findings highlight a range of standard structural and functional configurations in the microbial communities of a healthy population, paving the way for future research into the epidemiology, ecology, and translational applications of the human microbiome (Huttenhower et al., 2012).

These principles align with the CDP, which indicates the importance of a certain level of diversity for proper functionality. Thus, an increase or decrease in diversity is associated with various disease conditions.

**Figure 1:**
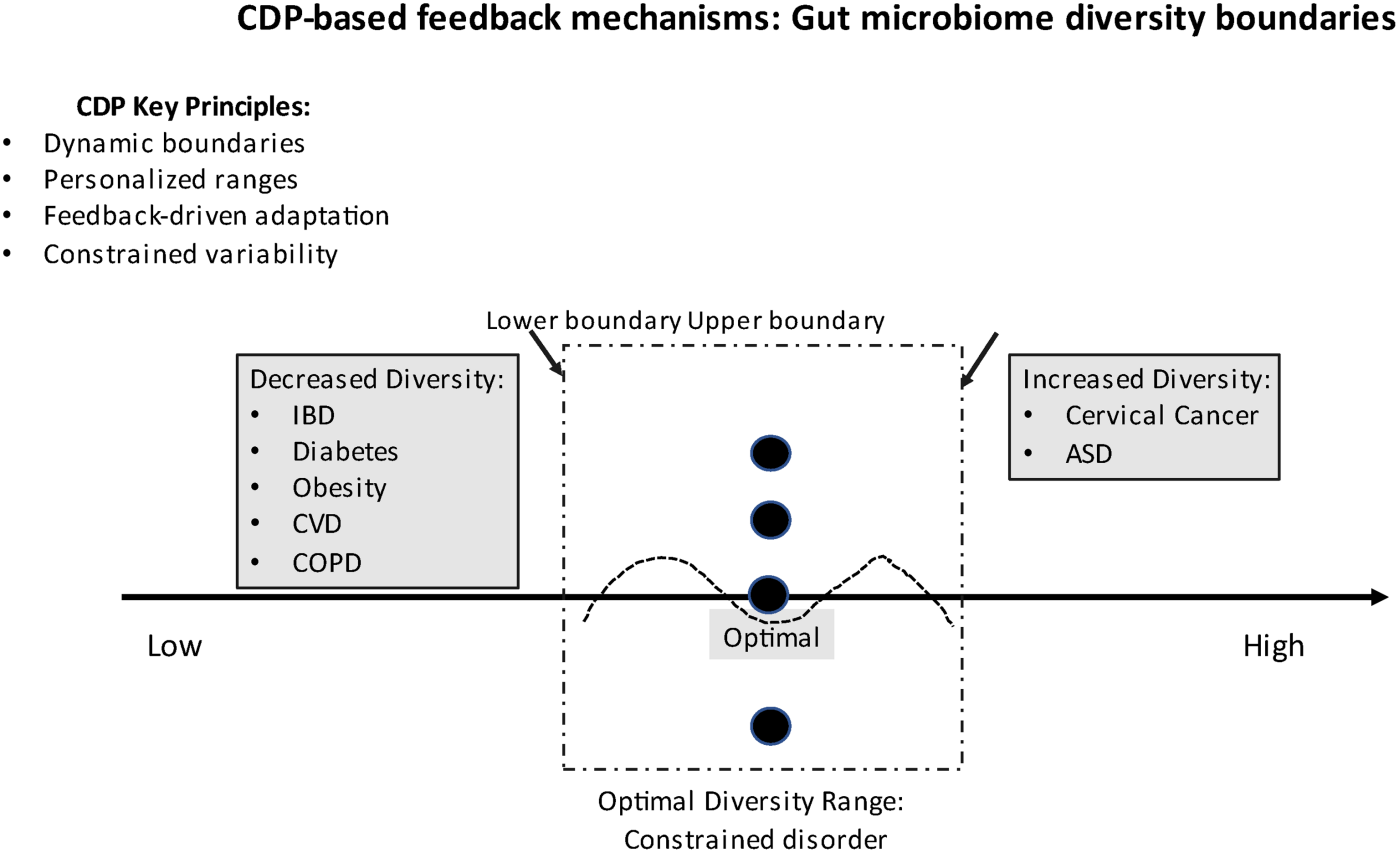
The CDP-based feedback mechanisms based on the gut microbiome diversity boundaries. Restoration and modulation mechanisms determine the range of the diversity. Central optimal diversity range (constrained disorder zone) with dynamic boundaries. Left zone: Decreased diversity associated with diseases (IBD, diabetes, obesity, CVD, COPD). Right zone: Increased diversity associated with conditions (cervical cancer, some ASD cases). Variability indicators within the optimal zone (dashed wave line).

Figure 1 illustrates the CDP-based feedback mechanisms and the concept of the active boundaries of the diversity of the microbiome.

## Genetic and environmental factors influence microbial diversity

Host genetics play a significant role in shaping microbial diversity and the overall composition and functionality of the microbiome. Specific gene variants that regulate immune responses, epithelial barrier integrity, and metabolic processes can contribute to variations in microbiome profiles. For example, genome-wide association studies (GWAS) have identified genetic loci, such as the lactase (LCT) gene, which is associated with the abundance of Bifidobacterium (Kurilshikov et al., 2021). Genetic factors can influence the gut environment, which impacts microbial colonization and diversity.

Age, sex, and hormonal status also significantly shape the gut microbiome. The gut microbiome evolves across various life stages, with early colonization influenced by maternal factors, delivery mode, and breastfeeding practices. During childhood, microbial diversity expands, stabilizing in adulthood before declining with age. Additionally, sex differences, influenced by hormonal variations, can create distinct microbial compositions between males and females. For instance, hormonal fluctuations during the menstrual cycle, pregnancy, and menopause can significantly impact the diversity and composition of gut microbiota (d'Afflitto et al., 2022; Jeong, 2022).

Environmental factors and lifestyle choices are equally crucial in determining microbial diversity. Diet is a primary modulator, with a Western diet—high in saturated fats and low in fiber—associated with reduced microbial diversity and increased pro-inflammatory species. Conversely, diets rich in fiber, polyphenols, and fermented foods promote the growth of beneficial bacteria that produce short-chain fatty acids, crucial for gut health (Illiano et al., 2020). Antibiotic use is another major disruptor, capable of eliminating entire bacterial populations, sometimes causing long-term dysbiosis. Non-antibiotic medications, such as proton pump inhibitors and NSAIDs, can alter microbial diversity. Likewise, chronic stress and poor sleep patterns can reduce microbial diversity, contributing to dysbiosis (Tasnim et al., 2017). Geography and lifestyle factors further influence microbial diversity. Populations in rural and non-industrialized societies typically harbor greater microbial diversity, likely due to increased exposure to diverse environmental microbes and a diet higher in unprocessed plant-based foods. Urbanization, reduced contact with nature, and higher sanitation standards are associated with lower microbial diversity (Kurilshikov et al., 2021).

These factors cause changes in microbial diversity closely connected to health and disease, emphasizing the need for specific, context-oriented strategies in microbiome research and therapy development (Hou et al., 2022; Shreiner et al., 2015).

## Challenges of current microbial interventions for recovering microbial diversity

Microbial interventions that aim to replicate and restore the diversity of a healthy gut microbiome have shown substantial promise in managing a wide range of disease states. Probiotics, live microorganisms that confer health benefits when consumed in adequate amounts, are widely used to restore microbial balance, enhance gut barrier function, and improve gut health. Certain probiotic strains have been found to modulate immune responses, alleviate symptoms of allergic disease, and reduce asthma severity. Recent advancements in genetic engineering have led to the development of tailored probiotics designed to enhance their functionality and survivability in the gut. These engineered probiotics show potential in targeting specific diseases by modulating immune responses and restoring microbial balance (Caldeira et al., 2020). However, the efficacy of probiotics can vary significantly between individuals, influenced by factors such as the composition of the baseline microbiome, diet, and genetic predispositions. It highlights the need for more personalized approaches.

Prebiotics are non-digestible food components that selectively enhance the growth and activity of beneficial gut bacteria, making them essential for improving microbial diversity. They contribute to microbial resilience, promote the production of short-chain fatty acids, and exhibit anti-inflammatory properties. When combined with probiotics as synbiotics, they can synergistically enhance their effectiveness and improve microbial engraftment (Ali et al., 2025).

Fecal microbiota transplantation (FMT) is an innovative and increasingly studied intervention involving transferring stool from a healthy donor to restore the patient’s microbial balance. Initially recognized for its effectiveness in treating *Clostridium difficile* infections, FMT is now being explored for its potential in managing inflammatory bowel disease (IBD), irritable bowel syndrome (IBS), and certain types of cancer (Caldeira et al., 2020; Chen et al., 2019). Despite advancements, challenges persist in donor selection, safety concerns, and variability in long-term outcomes, underscoring the need for optimized protocols and enhanced patient stratification.

Dietary interventions, particularly those rich in fiber, polyphenols, and plant-based foods, play a crucial role in shaping gut microbial communities. Specific nutrients, such as plant glycans, act as substrates for beneficial microbes, fostering the growth of commensal bacteria and enhancing microbiota resilience (Alexandrescu et al., 2025; Yaqub et al., 2025).

The data indicate that, although current microbial interventions hold substantial promise, further research and technological advancements are necessary to fully realize their potential. The CDP enables the creation of more adaptive, personalized therapies that leverage microbial diversity as a key element in health and disease management.

## Utilizing systems based on the Constrained-Disorder Principle to address the challenges of targeting gut diversity

Gut microbiome modeling, AI-driven microbiome analytics, and ecosystem stability have been explored over the last years for improving gut-diversity-based therapies (Alexandrescu et al., 2025; Dakal et al., 2025; Patil et al., 2025; Yaqub et al., 2025; Zhou and Zhao, 2025). AI systems are specifically designed to analyze microbiome data. These complex datasets can be accessed to extract valuable information using AI algorithms and machine learning techniques. By modeling gut microbial interactions, we can predict how microorganisms behave and their effects on host health and illness. AI can also assist in detecting microbial biomarkers, which are crucial indicators of gut health and potential disease risks. This innovative approach helps identify the root causes of diseases and facilitates the development of treatment strategies. Additionally, it enables personalized microbiome analysis, demonstrating how AI can assist individuals in making lifestyle adjustments tailored to their unique needs, ultimately enhancing their health (D’Urso and Broccolo, 2024; Patil et al., 2025).

The fact that both decreased and increased diversity in the gut microbiome can be linked to disease poses a significant challenge for targeting the microbiome as a therapeutic platform. Establishing what constitutes “healthy” microbial diversity is a considerable challenge, particularly given that the degree of diversity is dynamic and can vary under different environmental conditions. The complexity and diversity of the gut microbiome pose substantial challenges for its targeting in therapeutics (Ilan, 2019).

Biological systems utilize noise to better adapt to environmental challenges, emphasizing the importance of variability and stochastic gene expression in achieving robustness and adaptability. Applying CDP-based platforms may enable the identification of diversity ranges that balance ecosystem stability with functional adaptability. These ideal ranges are highly individualized and influenced by genetic, environmental, and lifestyle factors.

Second-generation AI systems offer unprecedented opportunities to operationalize the CDP. The approach focuses on a specific subject designed to improve patients’ clinical outcomes. A personalized, closed-loop system is introduced, designed to enhance end-organ function and improve the patient’s response to chronic therapies. This platform implements a tailored therapeutic regimen and incorporates measurable individualized variability patterns into its algorithm. The goal of this platform is to achieve clinically meaningful results by ensuring that chronic therapies have a sustainable effect while addressing the compensatory mechanisms linked to disease progression and drug resistance (Ilan, 2020a; Ilan, 2021a).

These systems may predict optimal microbial diversity ranges for individuals, simulate targeted interventions to fine-tune microbial ecosystems, and identify key biomarkers for disease prediction and personalized treatment. They leverage noise as a crucial component, selecting and targeting molecular and cellular disorders to enhance biological robustness (Adar et al., 2023; Azmanov et al., 2022; Azmanov et al., 2021; Gelman et al., 2020; Gelman et al., 2022; Hurvitz et al., 2021; Hurvitz et al., 2024; Hurvitz et al., 2022; Ilan, 2019; Ilan, 2021b; Ilan, 2023a; Ilan, 2024a–c; Ilan and Spigelman, 2020; Isahy and Ilan, 2021; Ishay et al., 2021a,b; Kenig and Ilan, 2019; Kenig et al., 2021; Kessler et al., 2020; Khoury and Ilan, 2019; Khoury and Ilan, 2021; Kolben et al., 2023; Kolben et al., 2021; Lehmann et al., 2023; Potruch et al., 2020; Sigawi et al., 2024b,c).

CDP-based algorithms have been utilized to develop resistance and drug tolerance in chronic diseases. It includes the development of resistance to multiple drugs over prolonged use, diuretic resistance in heart failure, drug-resistant cancer, immune-mediated diseases, and genetic disorders (Gelman et al., 2023; Hurvitz et al., 2024; Sigawi et al., 2024a; Sigawi et al., 2023).

CDP-based platforms are being developed in three stages. The first stage is an open-loop system that introduces variability into therapies within approved ranges. This approach can benefit targeting the gut microbiome by preventing tolerance and promoting higher degrees of diversity. It can also enhance the effectiveness of probiotics, prebiotics, and fecal microbiota transplantation (FMT) (Ilan, 2021a).

At the second level, a closed-loop system adjusts variability to predefined endpoints. It also customizes the algorithm’s output for each individual to enhance the response to intervention (Bayatra et al., 2024; Hurvitz and Ilan, 2023; Ilan, 2020b). The third level incorporates variability signatures into the algorithm to improve clinical outcomes. By generating personalized datasets from the CDP, valid biomarkers can be created to monitor disease progression, utilizing variability as a dynamic indicator (Sigawi et al., 2024c).

The results of the studies mentioned support the idea of using variability to improve effectiveness, but these findings are based on preliminary trials. To further evaluate the efficacy of these procedures, we need AI-based platforms that can personalize the output of these algorithms, as well as controlled trials. Regarding gut diversity, additional studies are necessary to determine the effectiveness of controlled variability and the range of variability in overcoming disease states. Current limitations in applying this approach in clinical settings include difficulties in quantifying variability ranges, ethical or practical barriers to individualized interventions, and a lack of comparative data.

The intermediate disturbance hypothesis suggests that disturbances can enhance biodiversity by creating diverse habitats (Dornelas, 2010). Moderate levels of stress, such as flooding, can lead to increased plant diversity through processes like erosion and sedimentation. It suggests that several common disturbances, such as antibiotic treatment, eating spicy food, and drinking water in unfamiliar environments, can be theoretically explained to some extent through the CDP (Dornelas, 2010).

In conclusion, the CDP provides a framework for redefining gut microbial diversity. With the advancements in artificial intelligence, this methodology can potentially transform microbiome research and clinical practices. It enables the development of precise and personalized interventions aimed at enhancing human health by using CDP-based AI systems that use gut diversity.
